# Supplementary material for: Cryopreservation of human cancers conserves tumour heterogeneity for single-cell multi-omics analysis
Source: Genome Med. 2021 May 10;13:81. doi: 10.1186/s13073-021-00885-z (PMC8111910; doi:10.1186/s13073-021-00885-z)
Supplement: Supplementary file 2 — Additional file 2: Supplementary Figures. Figures S1-S7. [file 13073_2021_885_MOESM2_ESM.pdf]

Figure S1

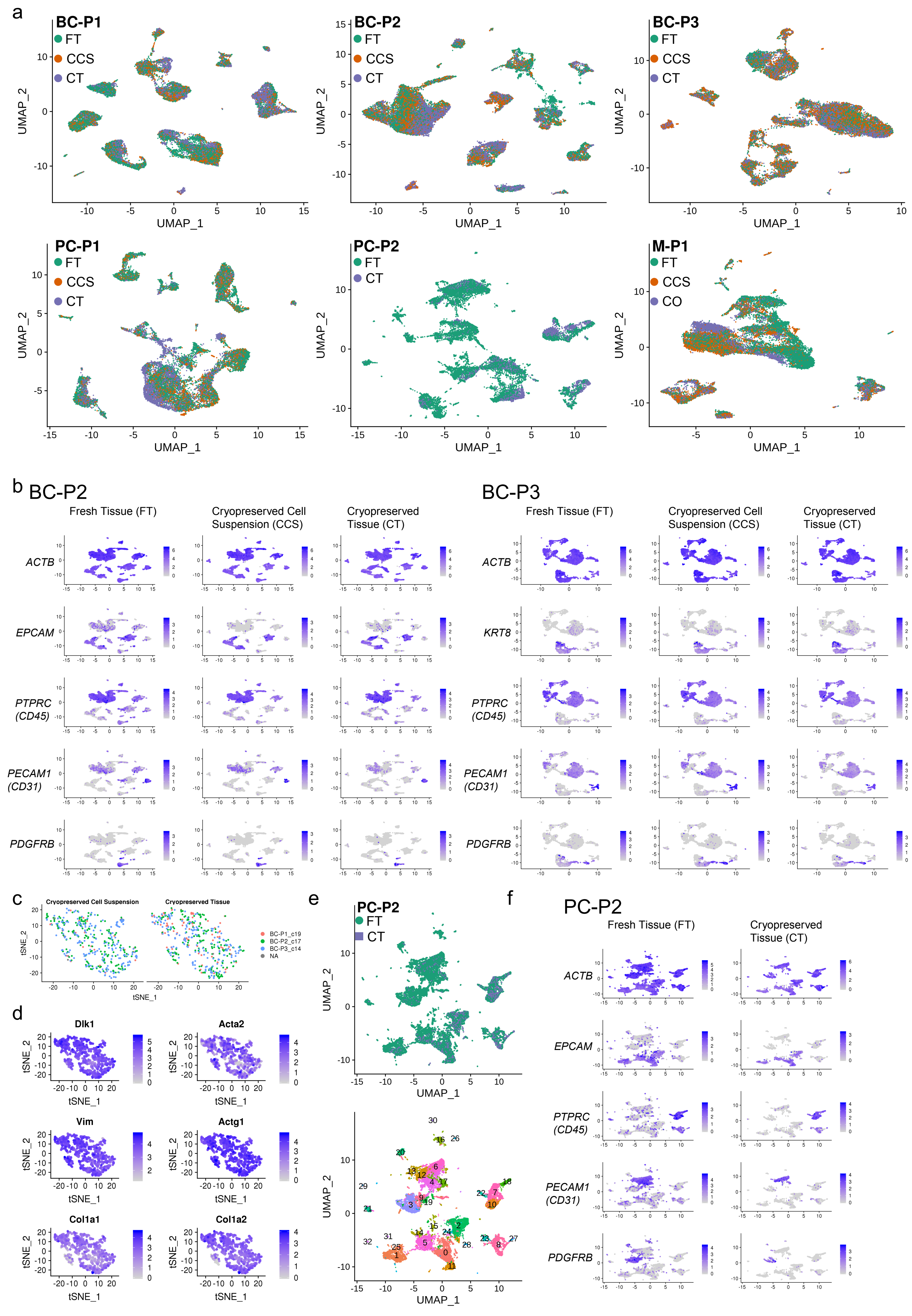
a
BC-P1
FT
CCS
CT
BC-P2
BC-P3
PC-P1
PC-P2
M-P1
CO
UMAP_1
UMAP_2
b
BC-P2
BC-P3
Fresh Tissue (FT)
Cryopreserved Cell Suspension (CCS)
Cryopreserved Tissue (CT)
ACTB
EPCAM
KRT8
PTPRC (CD45)
PECAM1 (CD31)
PDGFRB
c
Cryopreserved Cell Suspension
Cryopreserved Tissue
BC-P1_c19
BC-P2_c17
BC-P3_c14
NA
tSNE_1
tSNE_2
d
Dlk1
Acta2
Vim
Actg1
Col1a1
Col1a2
e
PC-P2
FT
CT
f
PC-P2
Fresh Tissue (FT)
Cryopreserved Tissue (CT)
ACTB
EPCAM
PTPRC (CD45)
PECAM1 (CD31)
PDGFRB

Figure S1. Cryopreservation allows for robust cell-type detection in clinical cancer samples. a, UMAP visualisations for the non-batch corrected data for each of the three breast cancer (BC-P1, BC-P2 and BC-P3), two prostate cancer (PC-P1 and PC-P2) and metastatic melanoma case (M-P1). **b,** Featureplot visualisations for additional breast cancer cases BC-P2 and BC-P3. Gene expression shows the conservation of the housekeeping gene *ACTB,* and markers for cancer/epithelial (*EPCAM*), immune (*PTPRC/*CD45), endothelial (*PECAM1*/CD31) and fibroblast/perivascular (*PDGFRB*) clusters following cryopreservation as CCS and CT. **c,** *t*SNE visualisation showing the high mixability of mouse NIH3T3 fibroblast cell line spike ins (~2%) from the cryopreserved replicates from all three breast cancer cases. Embeddings are split by cells captured from CCS and CT, respectively. Original cluster IDs from Figure 1b are c19 from BC-P1, c17 from BC-P2 and c14 from BC-P3. **d,** Featureplot visualisations of the NIH3T3 cell line fibroblast markers *Dlk1, Acta2, Vim, Actg1, Col1a1* and *Col1a2*. **e,** UMAP visualisations for the batch corrected data for PC-P2, which only contains comparisons between FT and CT replicates due to low cell numbers in the CCS replicate. UMAPs are coloured by cryopreserved conditions and cluster IDs. **f,** Featureplot visualisations of gene expression highlighting the conservation of the major cell lineages, as represented in **(b).**

Figure S2

a

BC-P1

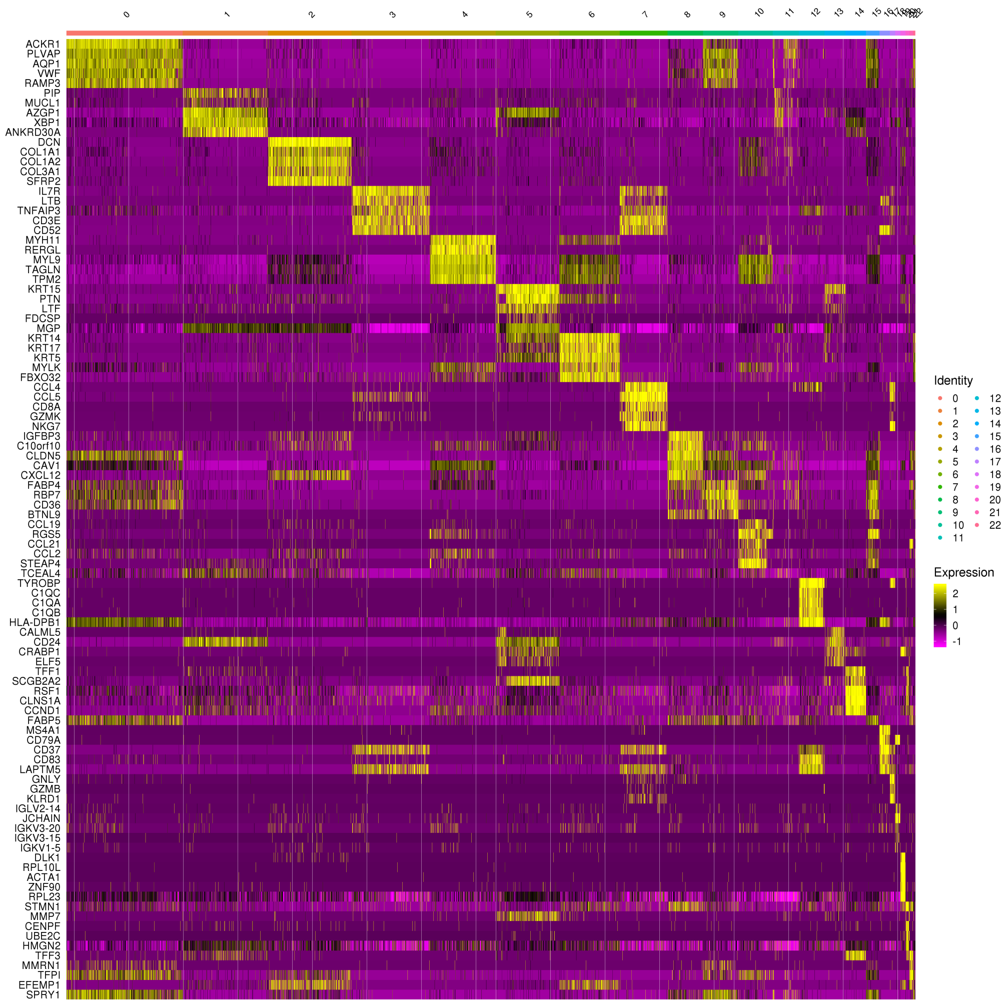

b

BC-P2

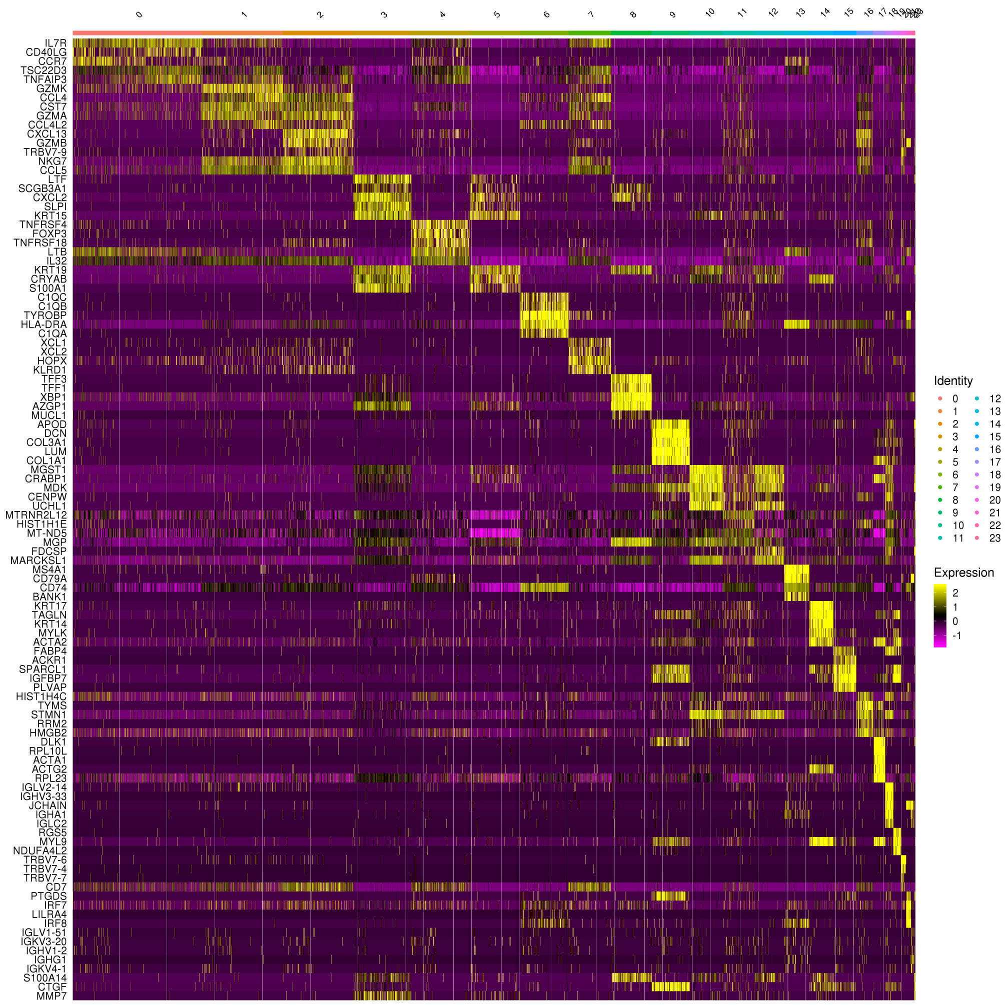

Figure S2 (cont.)

c

BC-P3

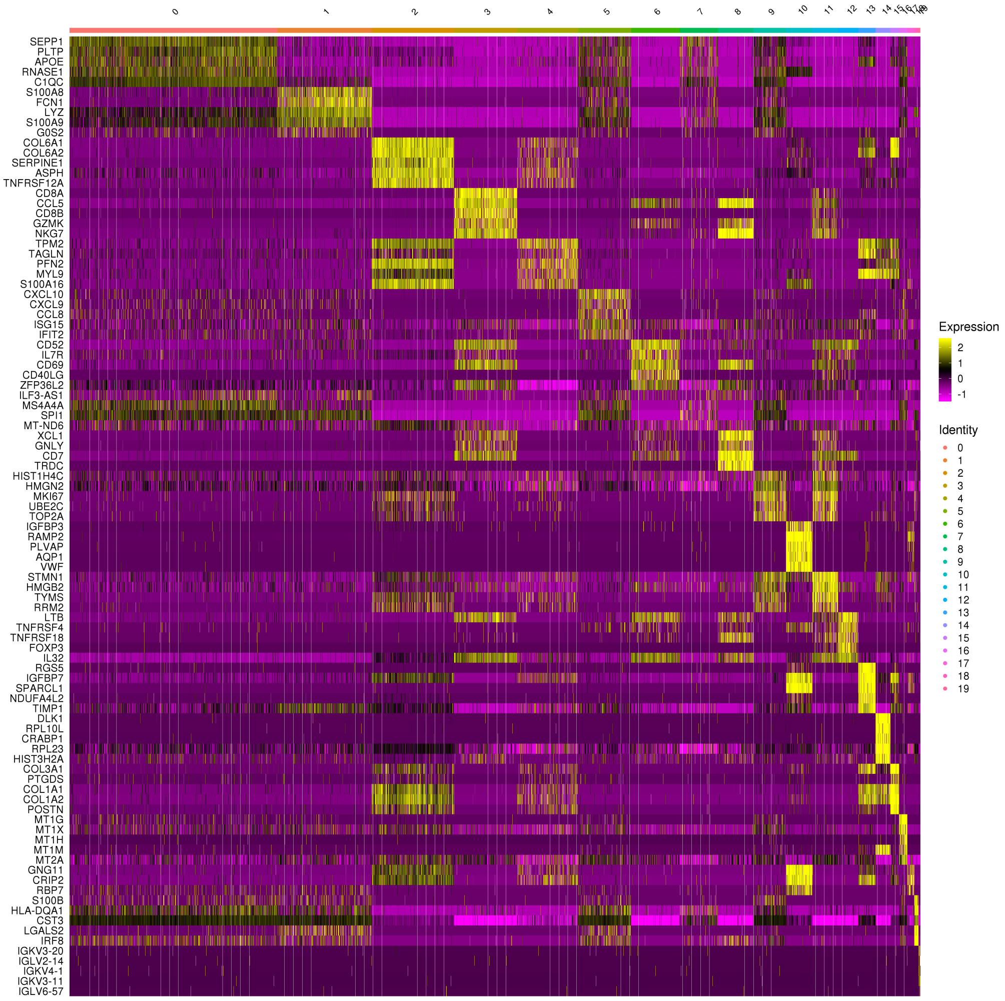

d

PC-P1

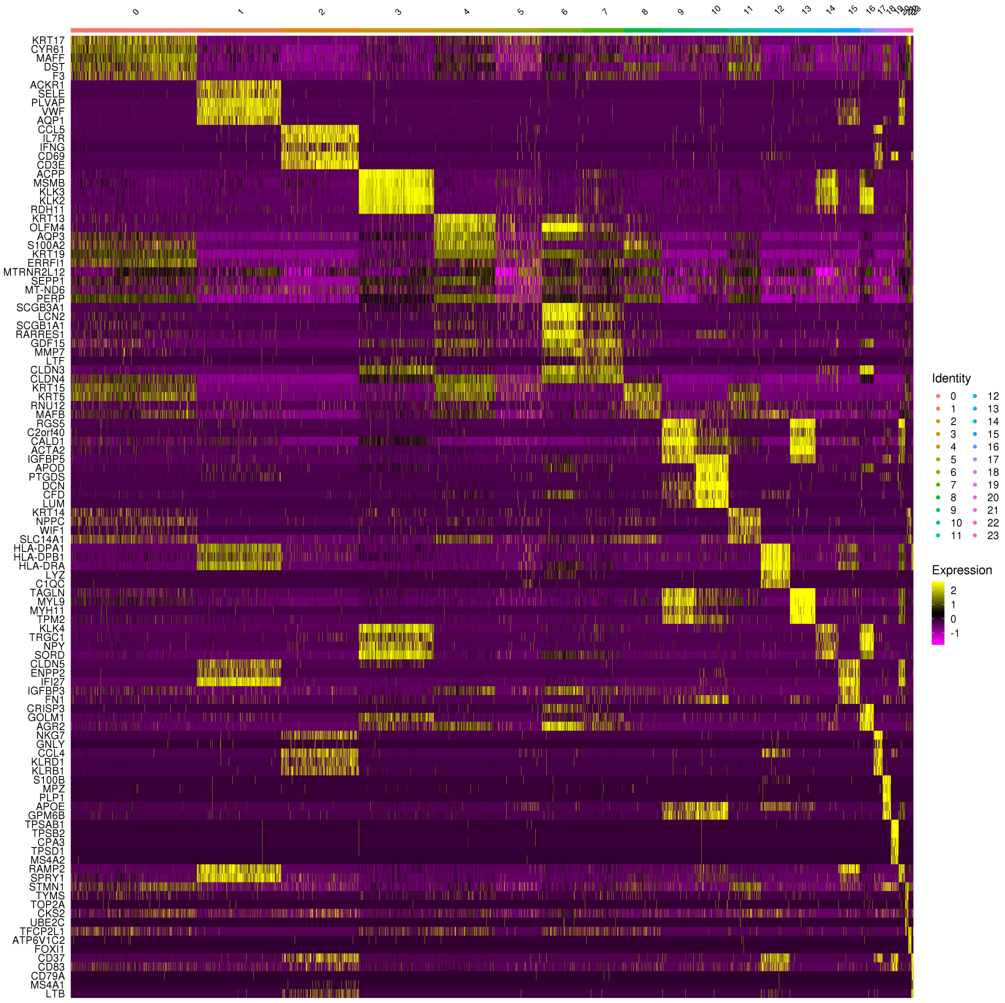

Figure S2 (cont.)

e

PC-P2

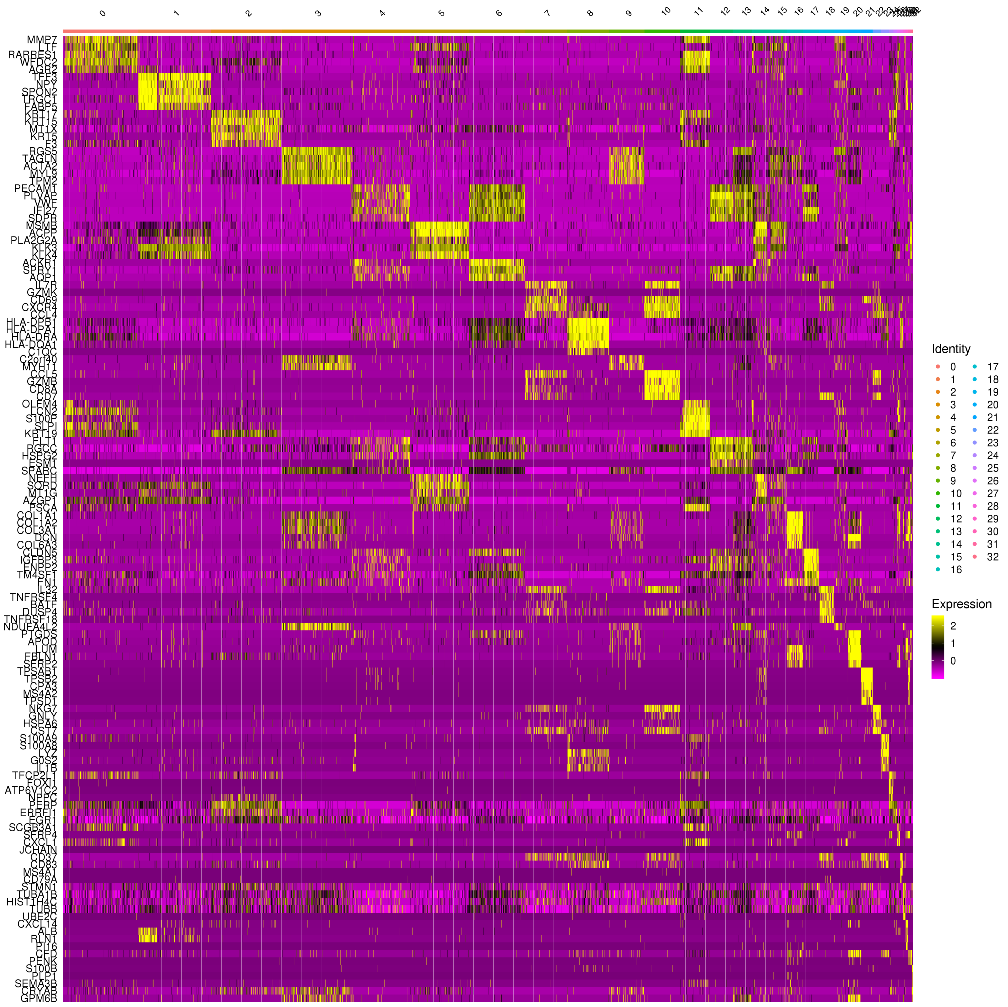

f

M-P1

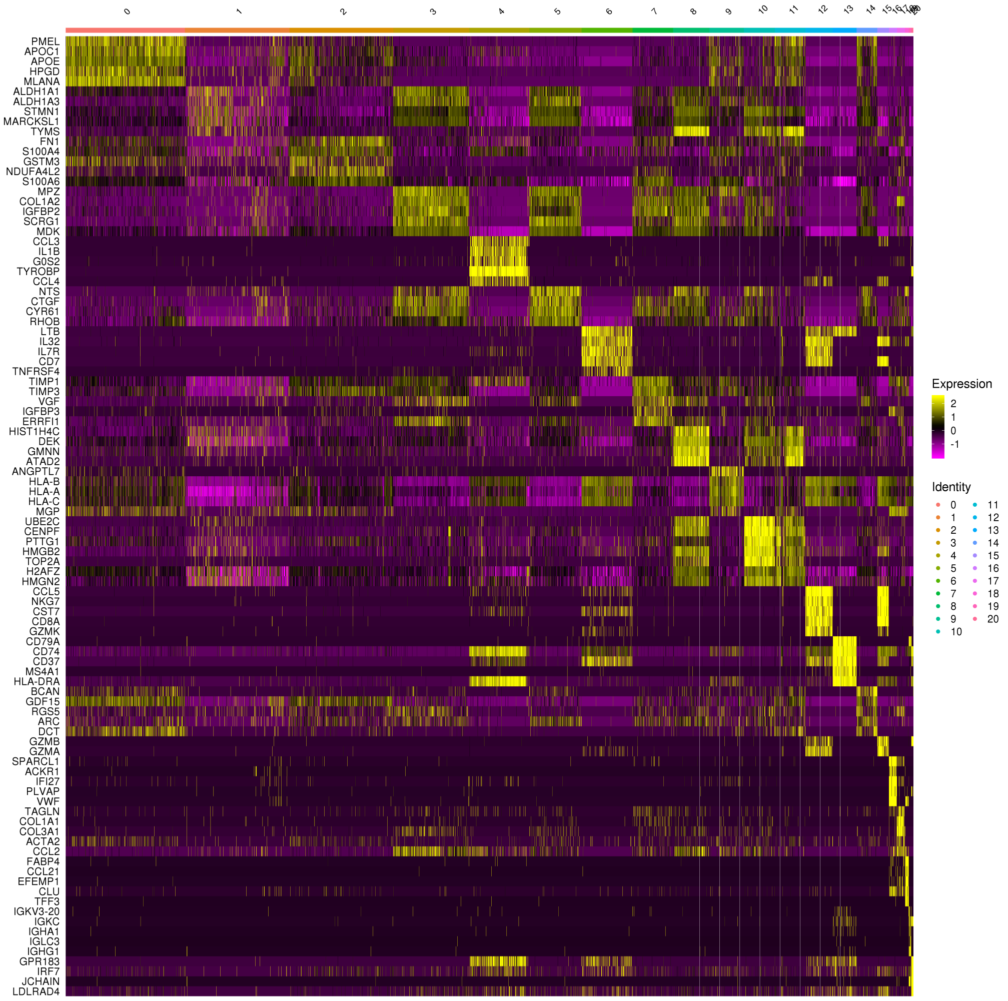

Figure S2. Heatmaps of integrated clusters for breast, prostate and melanoma cancer case. a-f, Heatmap visualisation of the top 5 differentially expressed genes per cluster for three breast cancer cases BC-P1 **(a)**, BC-P2 **(b)** and BC-P3 **(c)**, two prostate cancer cases PC-P1 **(d)** and PC-P2 **(e)** and a metastatic melanoma M-P1 (**f**). All cases represent the integrated clustering of all cryopreserved conditions. Differentially gene expression was performed using the MAST method within Seurat v3 with the RNA assay and default parameters. Heatmaps were generated using the DoHeatMap function using Seurat v3. Complete gene lists used are detailed in Supplementary Table 2.

Figure S3

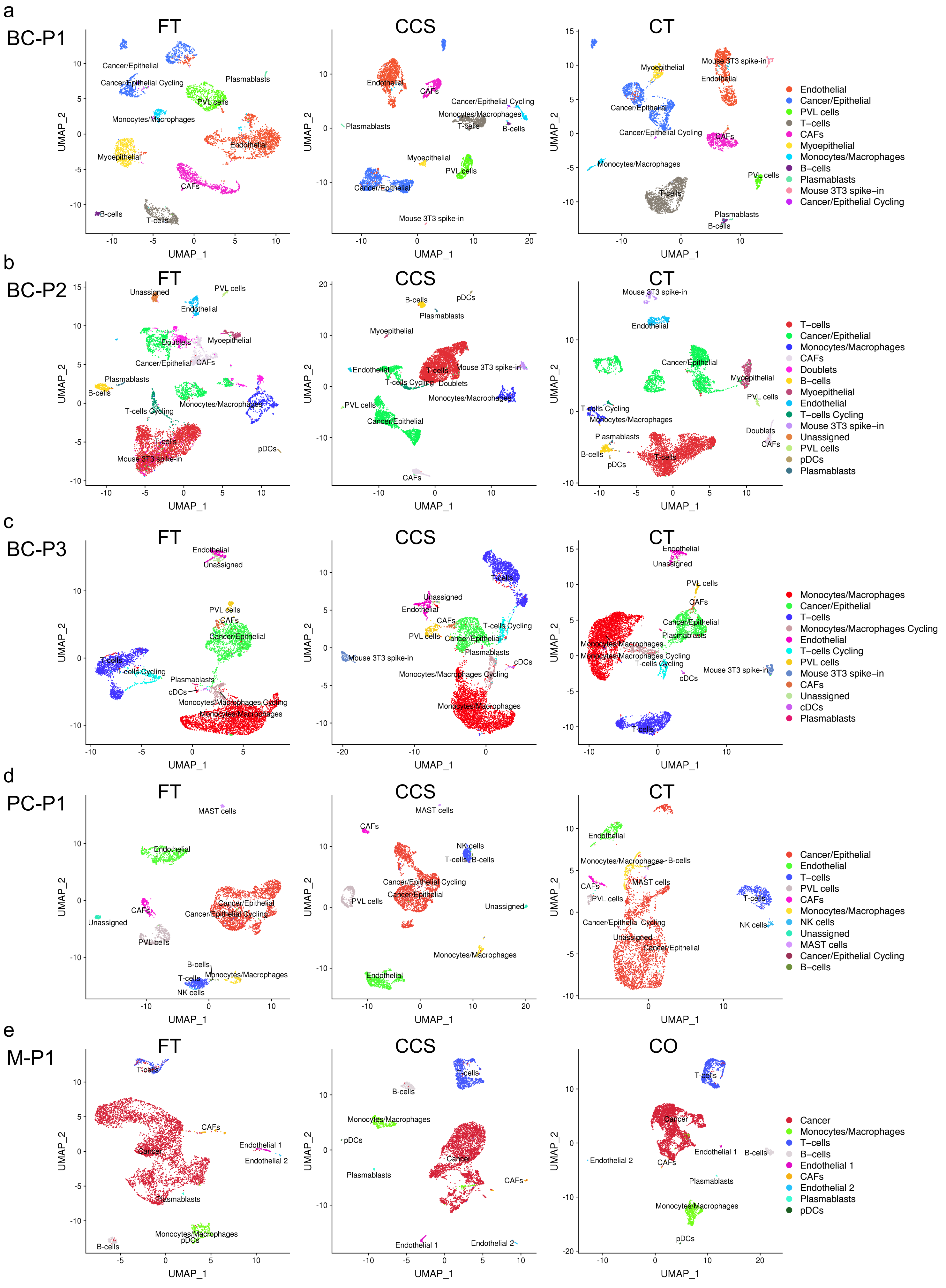
a
BC-P1
FT
CCS
CT
UMAP_1
UMAP_2
Endothelial
Cancer/Epithelial
PVL cells
T–cells
CAFs
Myoepithelial
Monocytes/Macrophages
B–cells
Plasmablasts
Mouse 3T3 spike–in
Cancer/Epithelial Cycling
b
BC-P2
FT
CCS
CT
T–cells
Cancer/Epithelial
Monocytes/Macrophages
CAFs
Doublets
B–cells
Myoepithelial
Endothelial
T–cells Cycling
Mouse 3T3 spike–in
Unassigned
PVL cells
pDCs
Plasmablasts
c
BC-P3
FT
CCS
CT
Monocytes/Macrophages
Cancer/Epithelial
T–cells
Monocytes/Macrophages Cycling
Endothelial
T–cells Cycling
PVL cells
Mouse 3T3 spike–in
CAFs
Unassigned
cDCs
Plasmablasts
d
PC-P1
FT
CCS
CT
Cancer/Epithelial
Endothelial
T–cells
PVL cells
CAFs
Monocytes/Macrophages
NK cells
Unassigned
MAST cells
Cancer/Epithelial Cycling
B–cells
e
M-P1
FT
CCS
CO
Cancer
Monocytes/Macrophages
T–cells
B–cells
Endothelial 1
CAFs
Endothelial 2
Plasmablasts
pDCs

Figure S3. Unbiased clustering and cluster annotation in individual fresh tissue and cryopreserved samples. UMAP visualisations for individual fresh tissue (FT), cryopreserved cell suspension (CCS), cryopreserved tissue (CT) and cryopreserved overnight (CO) samples for the breast cancer cases BC-P1 (**a**), BC-P2 (**b**) and BC-P3 (**c**), prostate cancer case PC-P1 (**d**) and metastatic melanoma case M-P1 (**e**). All matched individual datasets were down sampled to the lowest replicate cell number and analysed independently using unbiased clustering with the default resolution of 0.8 and UMAP dimensionality reduction with the top 20 principal components. Cell annotations plotted are derived from the integrated datasets as in Figure 1b and Figure 2a-b.

Figure S4

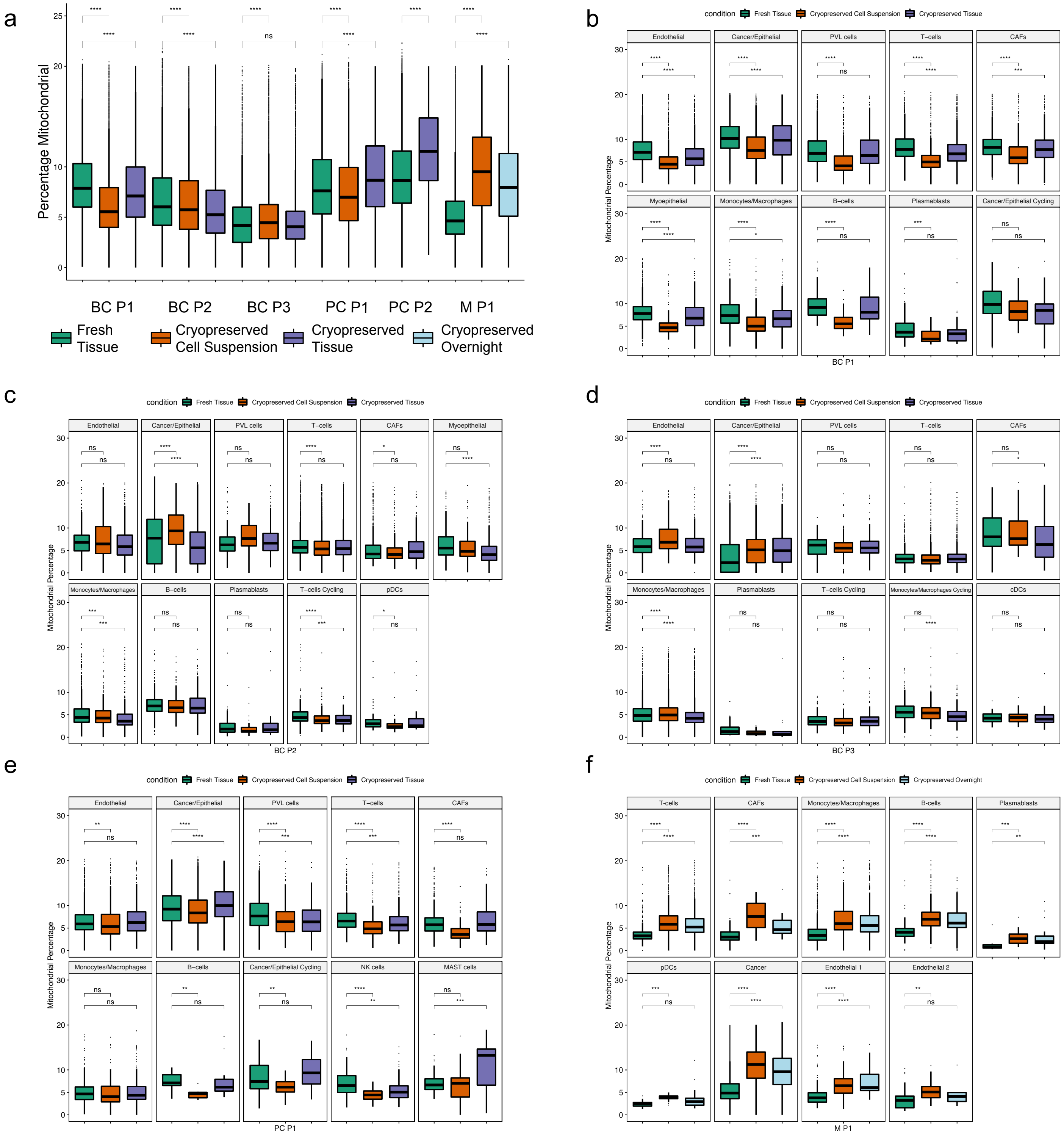
a
b
c
d
e
f
Percentage Mitochondrial
Mitochondrial Percentage
BC P1
BC P2
BC P3
PC P1
PC P2
M P1
Fresh Tissue
Cryopreserved Cell Suspension
Cryopreserved Tissue
Cryopreserved Overnight
condition
Endothelial
Cancer/Epithelial
PVL cells
T−cells
CAFs
Myoepithelial
Monocytes/Macrophages
B−cells
Plasmablasts
Cancer/Epithelial Cycling
T−cells Cycling
pDCs
Monocytes/Macrophages Cycling
cDCs
NK cells
MAST cells
Cancer
Endothelial 1
Endothelial 2

Figure S4. Percentage of mitochondrial transcripts across cryopreservation conditions and cell types. a-f, Percentage of mitochondrial genes per cell across cryopreservation conditions: Fresh Tissue (FT; green), Cryopreserved Cell Suspension (CCS; orange), Cryopreserved Tissue (CT; purple) and Cryopreserved Overnight (CO; light blue). Comparisons are plotted across all cells (**a**) and faceted by annotated cell type for each of the tumour cases analysed BC-P1 (**b**), BC-P2, (**c**), BC-P3 (**d**), PC-P1 (**e**) and M-P1 (**f**). Statistical significance was computed using an unpaired wilcoxon test where P-values denoted by asterisks: *p < 0.05, p < 0.01, *p < 0.001 and ****p < 0.0001.

Figure S5

a

BC-P1

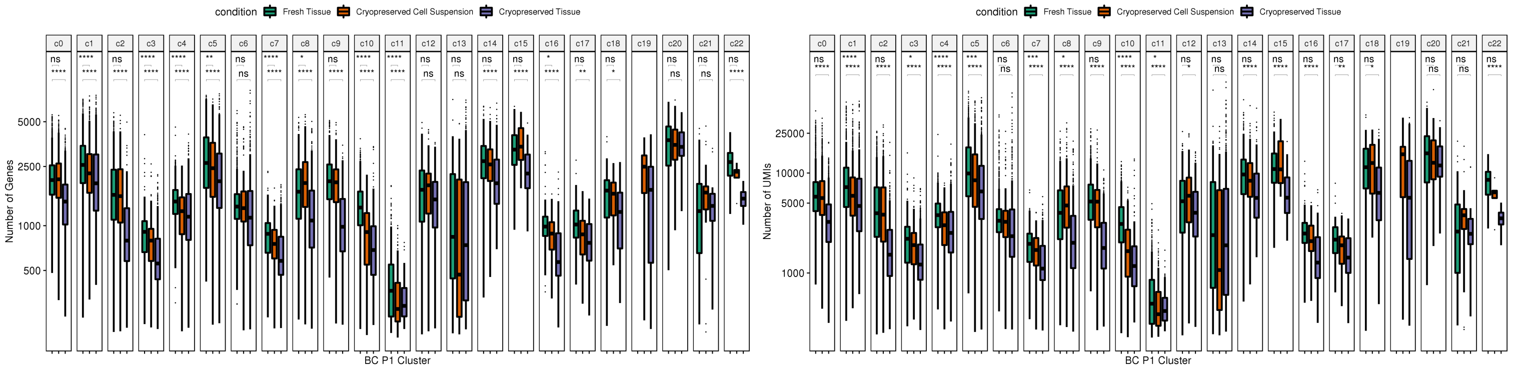

b

BC-P2

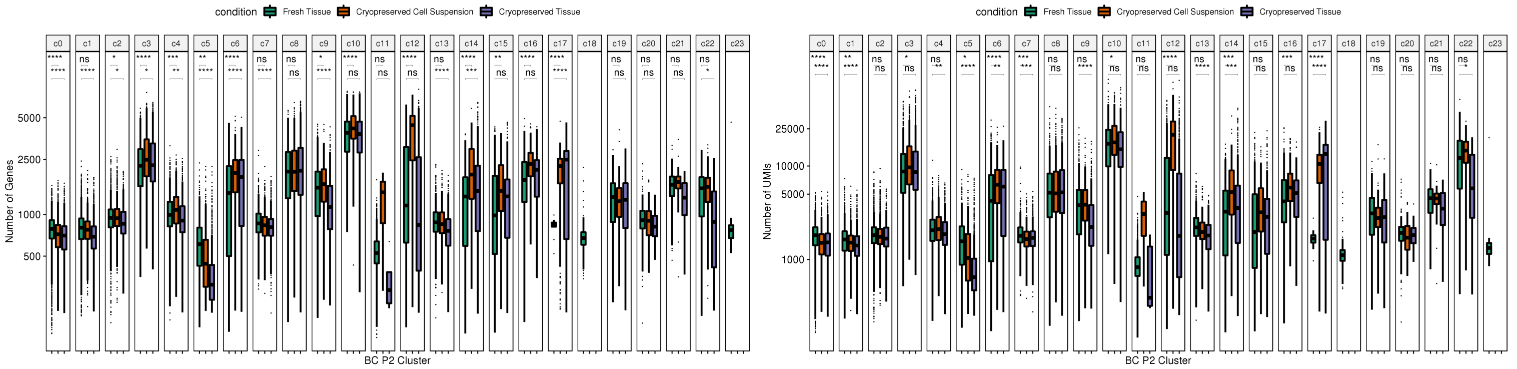

c

BC-P3

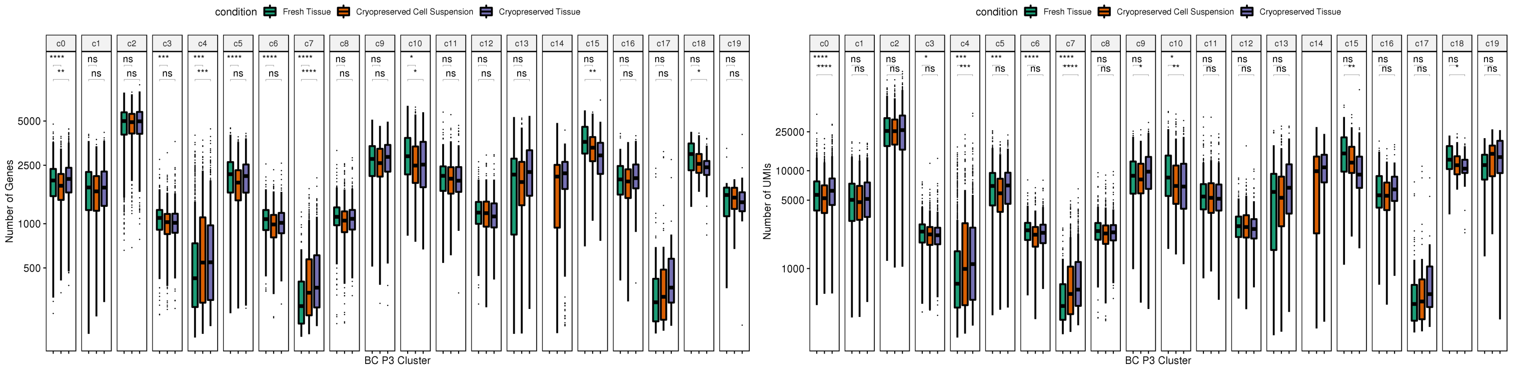

d

PC-P1

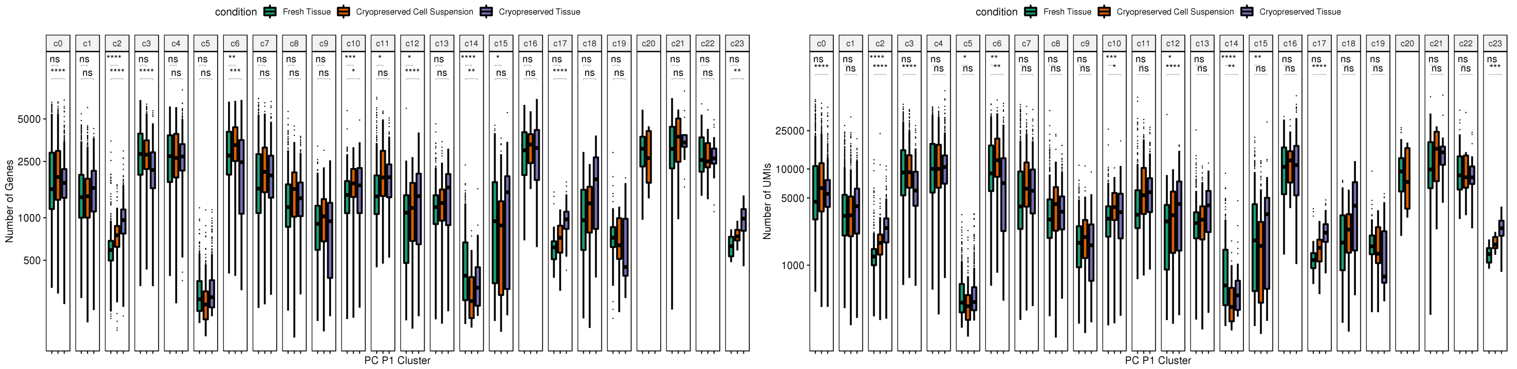

Figure S5 (cont.)

e

BC-P2

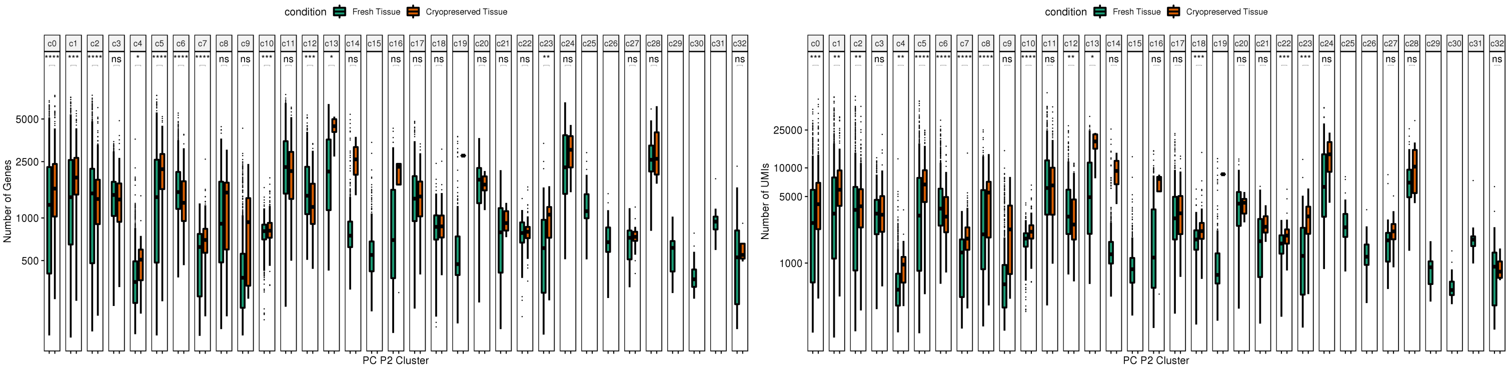

f

M-P1

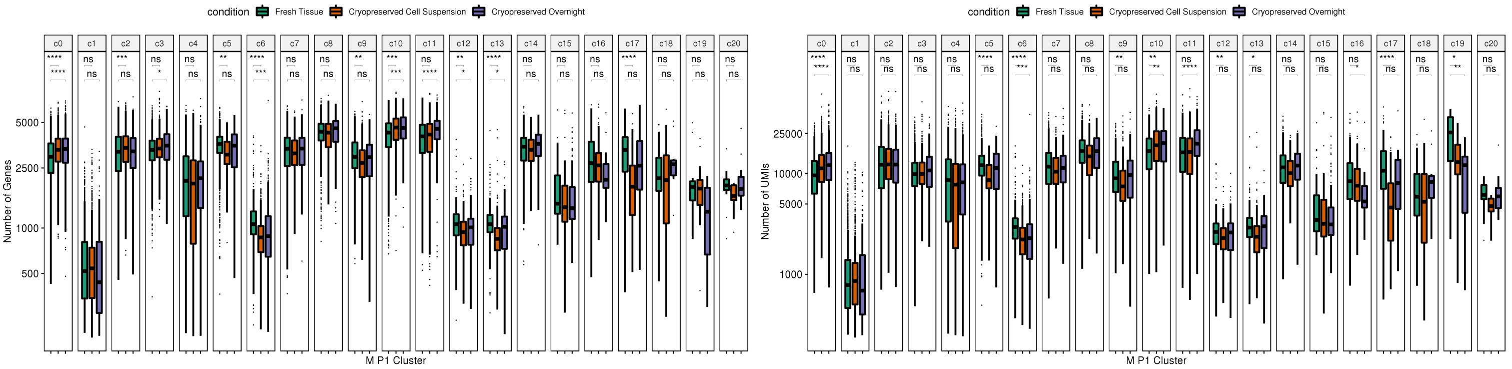

Figure S5. Number of genes and UMIs per cluster. a-f, Number of genes (left) and UMIs (right) detected per cell per cluster across FT (green), CCS (orange), CT (purple) and CO (purple; melanoma case only) replicates of breast cancer cases BC-P1 **(a)**, BC-P2 **(b)** and BC-P3 **(c)**, prostate cancer cases PC-P1 **(d)** and PC-P2 (**e**) and a metastatic melanoma M-P1 (**f**). Sequencing libraries were down sampled to equal number of mapped reads per cell using cellranger aggregate function to account for differences from sequencing depth. Statistical significance was determined using an unpaired Student's *t*-test.

Figure S6

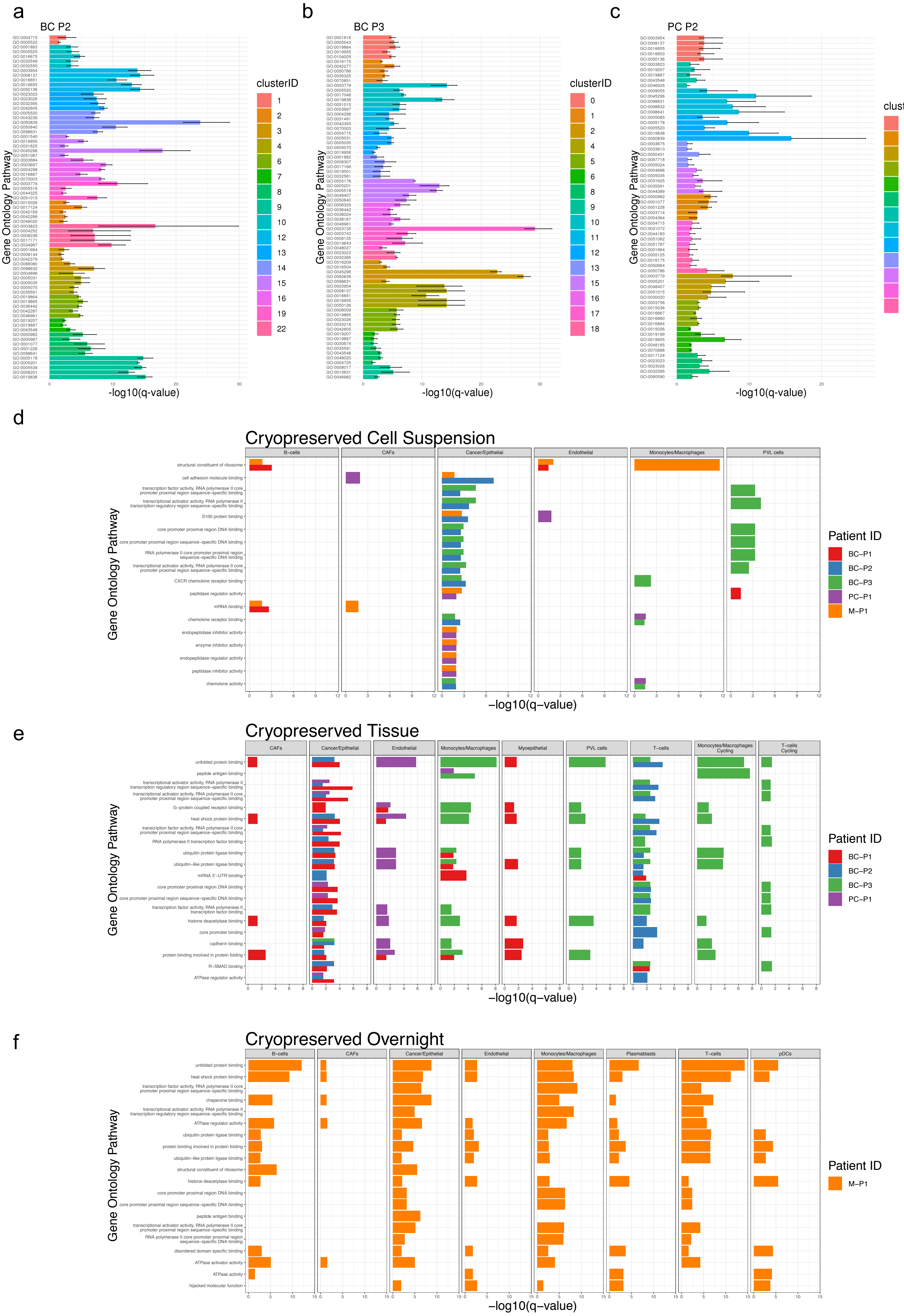

a
BC P2
b
BC P3
c
PC P2
Gene Ontology Pathway
-log10(q-value)
clusterID
d
Cryopreserved Cell Suspension
B−cells
CAFs
Cancer/Epithelial
Endothelial
Monocytes/Macrophages
PVL cells
structural constituent of ribosome
cell adhesion molecule binding
transcription factor activity, RNA polymerase II core promoter proximal region sequence−specific binding
transcriptional activator activity, RNA polymerase II transcription regulatory region sequence−specific binding
S100 protein binding
core promoter proximal region DNA binding
core promoter proximal region sequence−specific DNA binding
RNA polymerase II core promoter proximal region sequence−specific DNA binding
transcriptional activator activity, RNA polymerase II core promoter proximal region sequence−specific binding
CXCR chemokine receptor binding
peptidase regulator activity
mRNA binding
chemokine receptor binding
endopeptidase inhibitor activity
enzyme inhibitor activity
endopeptidase regulator activity
peptidase inhibitor activity
chemokine activity
−log10(q−value)
Patient ID
BC−P1
BC−P2
BC−P3
PC−P1
M−P1
e
Cryopreserved Tissue
CAFs
Cancer/Epithelial
Endothelial
Monocytes/Macrophages
Myoepithelial
PVL cells
T−cells
Monocytes/Macrophages Cycling
T−cells Cycling
unfolded protein binding
peptide antigen binding
transcriptional activator activity, RNA polymerase II transcription regulatory region sequence−specific binding
transcriptional activator activity, RNA polymerase II core promoter proximal region sequence−specific binding
G−protein coupled receptor binding
heat shock protein binding
transcription factor activity, RNA polymerase II core promoter proximal region sequence−specific binding
RNA polymerase II transcription factor binding
ubiquitin protein ligase binding
ubiquitin−like protein ligase binding
mRNA 3'−UTR binding
core promoter proximal region DNA binding
core promoter proximal region sequence−specific DNA binding
transcription factor activity, RNA polymerase II transcription factor binding
histone deacetylase binding
core promoter binding
cadherin binding
protein binding involved in protein folding
R−SMAD binding
ATPase regulator activity
−log10(q−value)
Patient ID
BC−P1
BC−P2
BC−P3
PC−P1
f
Cryopreserved Overnight
B−cells
CAFs
Cancer/Epithelial
Endothelial
Monocytes/Macrophages
Plasmablasts
T−cells
pDCs
unfolded protein binding
heat shock protein binding
transcription factor activity, RNA polymerase II core promoter proximal region sequence−specific binding
chaperone binding
transcriptional activator activity, RNA polymerase II transcription regulatory region sequence−specific binding
ATPase regulator activity
ubiquitin protein ligase binding
protein binding involved in protein folding
ubiquitin−like protein ligase binding
structural constituent of ribosome
histone deacetylase binding
core promoter proximal region DNA binding
core promoter proximal region sequence−specific DNA binding
peptide antigen binding
transcriptional activator activity, RNA polymerase II core promoter proximal region sequence−specific binding
RNA polymerase II core promoter proximal region sequence−specific DNA binding
disordered domain specific binding
ATPase activator activity
ATPase activity
hijacked molecular function
−log10(q−value)
Patient ID
M−P1

Figure S6. Cryopreservation maintains the detection of biological pathways in additional cases. a-c, Sensitivity of pathway enrichment scores detected in clusters across cryopreserved replicates. Additional representative cases of breast cancer BC-P2 **(a)** and BC-P3 (**b**) and prostate cancer PC-P2 (**c**) are shown. The minimum, mean and maximum -log10 q-value are plotted in the error bars of each GO pathway. All DEGs from each cluster were passed on to the ClusterProfiler package for functional enrichment with the CC sub-ontology under the human org.Hs.eg.db database. GO pathway descriptions can be found in Supplementary Table 3. **d-f**, Enrichment scores for gene ontology pathways that are unique to cryopreservation conditions for each annotated cell type: cryopreserved cell suspension (CCS; **d**), cryopreserved tissue (CT; **e**) and cryopreserved cell suspension after overnight cold storage (CO; **f**). Comparisons were performed for each annotated cell type from each matched condition, which were first down sampled by total cell number and total number of sequencing reads per sample. For the CCS (**d**) and CT (**e**) conditions, only pathways that were shared across multiple cases were analysed. Only the top 10 pathways based on enrichment scores are plotted. DEGs from each cell type and condition (Supplementary Table S6) were passed on to the ClusterProfiler package for functional enrichment with the CC sub-ontology under the human org.Hs.eg.db database. All GO pathway descriptions can be found in Supplementary Table S7.

Supplementary Figure 7

a

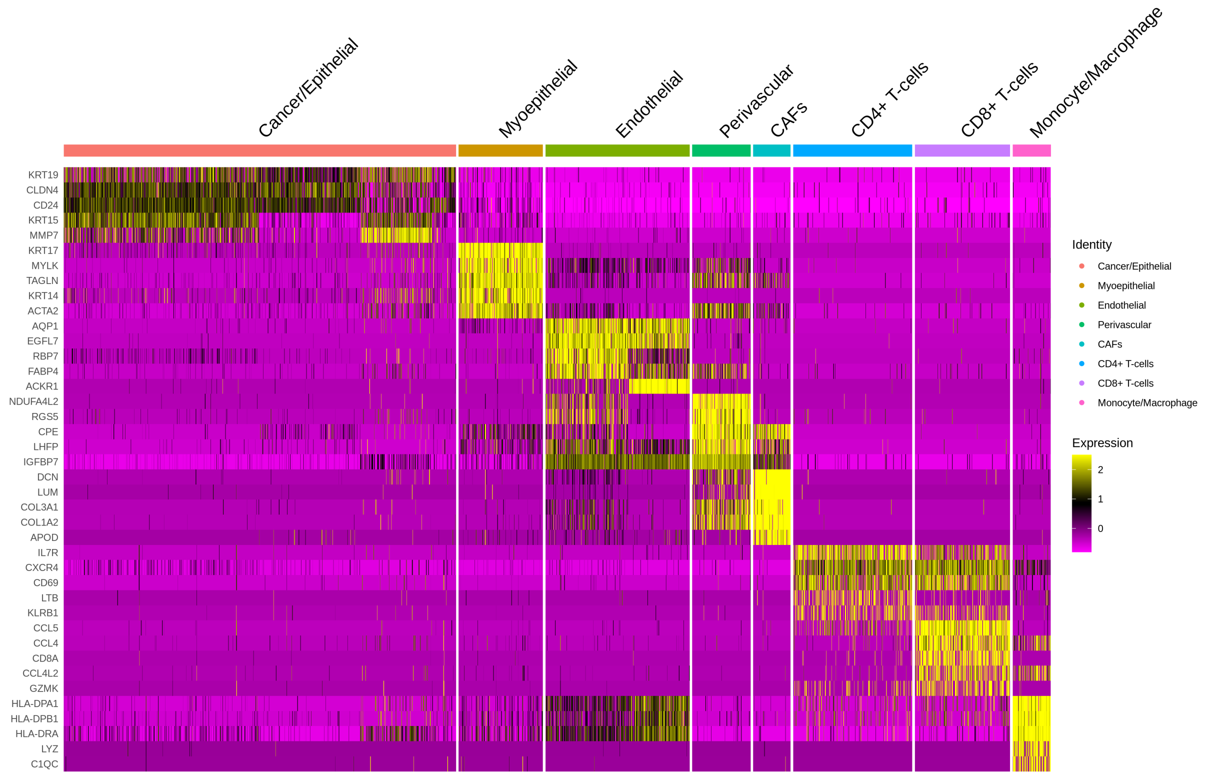

b

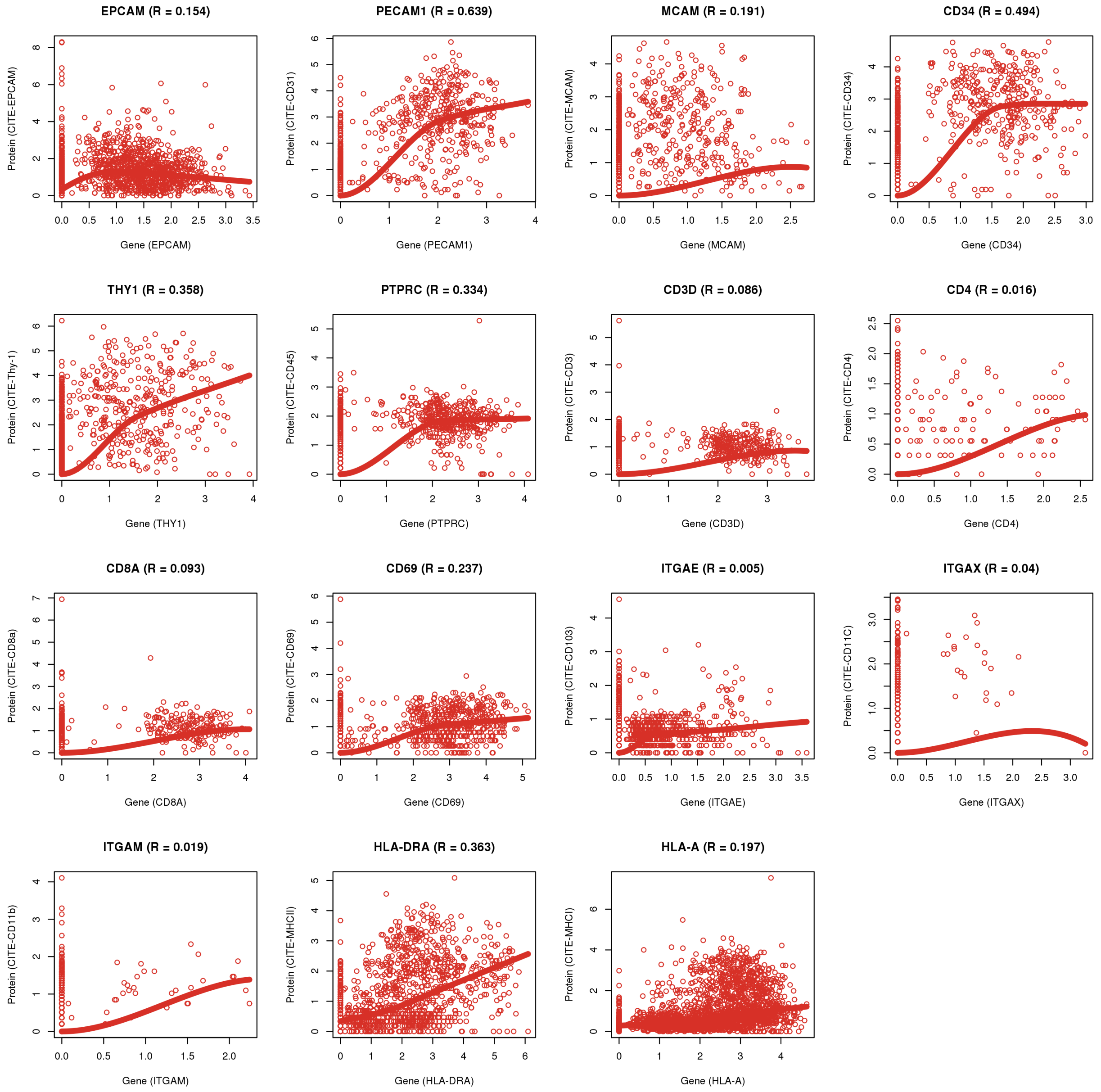

Figure S7. Cryopreservation provides high quality immunophenotyping using CITE-Seq. **a,** Heatmap visualisation of the top 5 differentially expressed genes of indicated canonical cell type markers for an independent breast cancer case for the CITE-Seq experiment. Differentially gene expression was performed using the MAST method within Seurat v3 with the RNA assay and default parameters. Heatmaps were generated using the DoHeatMap function using Seurat v3. **b,** Correlation plots between protein and genes for the panel of 15 markers used for CITE-Seq. Correlation values (adjusted-R^2) were computed using linear regression in *R* to model the log-normalised gene expression value and corresponding ADT levels.
